# Gate Alignment of Liquid Water Molecules in Electric Double Layer

**DOI:** 10.3389/fchem.2021.717167

**Published:** 2021-08-17

**Authors:** Xiaoqun Li, Xin Lin, Ying Li, Wei-Tao Liu

**Affiliations:** Physics Department, State Key Laboratory of Surface Physics, Key Laboratory of Micro and Nano Photonic Structures [Ministry of Education (MOE)], Fudan University, Shanghai, China

**Keywords:** oxide interface, water interface, electric double layer, electrochemical gating, sum frequency vibrational spectroscopy, field alignment of water

## Abstract

The behavior of liquid water molecules near an electrified interface is important to many disciplines of science and engineering. In this study, we applied an external gate potential to the silica/water interface via an electrolyte-insulator-semiconductor (EIS) junction to control the surface charging state. Without varying the ionic composition in water, the electrical gating allowed an efficient tuning of the interfacial charge density and field. Using the sum-frequency vibrational spectroscopy, we found a drastic enhancement of interfacial OH vibrational signals at high potential in weakly acidic water, which exceeded that from conventional bulk-silica/water interfaces even in strong basic solutions. Analysis of the spectra indicated that it was due to the alignment of liquid water molecules through the electric double layer, where the screening was weak because of the low ion density. Such a combination of strong field and weak screening demonstrates the unique tuning capability of the EIS scheme, and would allow us to investigate a wealth of phenomena at charged oxide/water interfaces.

## Introduction

The oxides/water interfaces are ubiquitous in nature, and are important to an enormous range of fields including environmental science, geoscience, catalysis, etc (Putnis, 2014; Covert and Hore, 2016; Schaefer et al., 2018). At these interfaces, oxide surface lattices react with vicinal water molecules, forming charged surface groups that may only be stable in aqueous environments, and lead to the formation of electric double layers (EDL) (Brown, 2001; Gaigeot et al., 2012; Sulpizi et al., 2012; Brown et al., 2016; Boily et al., 2019). The rich phenomena and functionalities of oxide/water interfaces are largely based on their interfacial charging states and properties of EDLs (Brown, 2001; Putnis, 2014; Covert and Hore, 2016; Ohno et al., 2016; Schaefer et al., 2018). For example, the water electric dipole moment can be aligned by the electrostatic field in EDL and affect the interfacial chemistry (Brown, 2001; Nihonyanagi et al., 2004; Aragones et al., 2011; Saitta et al., 2012; Brown et al., 2016; Montenegro et al., 2021). In the case of ice, such field alignment could give rise to a ferroelectric state of highly oriented water molecules, which was readily probed using the surface-sensitive sum-frequency vibrational spectroscopy (SFVS) (Miranda et al., 1998; Verdaguer et al., 2006; Anim-Danso et al., 2016). Whether similar states exist or not in the liquid phase has remained unclear. One problem is that, to achieve a strong polar ordering of water in EDLs, it requires a large electrostatic field thus a high surface charge density on the solid surface. Meanwhile, the surface charge density was usually tuned through protonation/deprotonation of surface lattices by varying the water pH value (Brown et al., 2016). Unfortunately, many oxide/water interfaces have their point-zero-charge (pzc) near neutral pH (Kosmulski, 2002; Backus et al., 2020), hence cannot reach very high surface charge density. The amorphous silicon dioxide (SiO_2_, silica) in water has a low pzc value of around pH 2∼3, thus can reach a high density of negative charges in strong basic solutions. However, the ion concentration in water is then inevitably large, which screens the surface field and reduces the EDL thickness (Jena et al., 2011; DeWalt-Kerian et al., 2017). In our previous study (Wang et al., 2019), we demonstrated a new scheme to control the oxide surface charging state utilizing an electrolyte-insulator-semiconductor (EIS) junction (Waleed Shinwari et al., 2007). The scheme allowed us to tune the surface charge and field without varying the electrolyte ionic composition, which provided a new dimension to control the charged solid/liquid interfaces. Nonetheless, the native oxide used before withstood only small gate potential and exhibited large leak current due to defects, which limited its tunability of the surface charging state.

In the present work, we utilized a much thicker SiO_2_ film of ∼100 nm that allowed the application of +/−50 V of gate potential, in contrast to the +/−1 V in the previous case. The thicker film also had a much smaller capacitance, leading to a much greater potential drop and stronger electric polarization across the film, as well as a higher net surface charge density. We used SFVS to investigate OH stretching vibrational spectra from the SiO_2_ interface in neutral water (exposed to the atmosphere). From zero potential to −50 V on the silicon electrode, we observed a drastic enhancement of the interfacial OH signal, which even exceeded that observed at the bulk-silica/water interfaces in strong basic solutions (Ostroverkhov et al., 2004, 2005; DeWalt-Kerian et al., 2017). The silica/water interface can be strongly negatively charged at high pH, which supposedly would induce a highly ordered interfacial water structure (Ostroverkhov et al., 2004; 2005). The greater OH intensity in our case suggested that external gating can lead to an even higher degree of polar ordering in the OH structure. Toward more positive gate potential, the OH signal dropped asymmetrically, exhibiting similar spectral profiles to those from bulk-silica/water interfaces at low pH values. Combined with fitting results, we attributed the observation to an effective field alignment of water molecules inside the diffuse layer. Because the ion density remained low in our case, the electrostatic screening of the surface field was poor, and the long-range alignment of water molecules could be readily detected. Basically, our study demonstrated the unique tuning capability of aqueous oxide interfaces by externally gating the EIS junction, and allowed us to achieve an overall greater polar alignment of interfacial water molecules compared to that achievable *via* conventional means.

## Materials and Methods

### Sample and Chemicals

**FIGURE 1:**
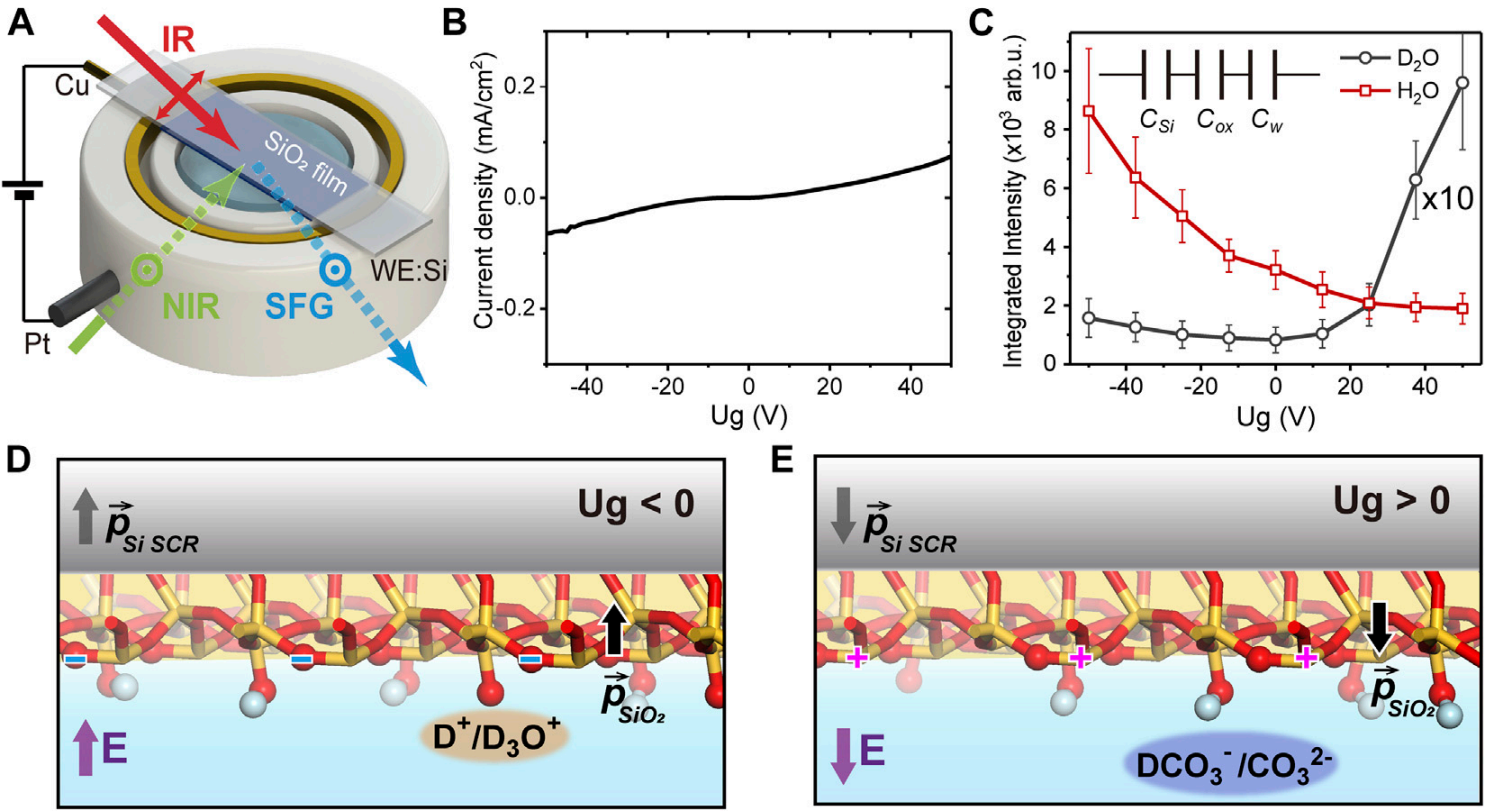
**(A)** Sketch of the experimental geometry. **(B)** The leak current versus the gate potential (*U*_*g*_). **(C)** The gate-dependent spectrally-integrated SFVS signal in D_2_O (black, magnified by 10 times) and H_2_O (red). Inset: the capacitor-in-series model of the junction. Sketches of the interfacial structure in D_2_O experiments at **(D)** *U*_*g*_ *< 0* and **(E)** *U*_*g*_ *> 0*.

The experimental and sample geometries were sketched in Figure 1A. The silicon (Si) substrate was in contact with a copper ring as the gate electrode, and a platinum wire in water serving as the ground (GND). A silicon dioxide (SiO_2_) thin film of about ∼100 nm-thick was deposited through magnetron sputtering procedure (DE500 Sputter) on the silicon wafer (0.5 mm-thick Si (100), n-doped, resistivity: 1–10 Ω cm). A source meter (Keithley 2400) was used to control the gate potential (*U*_*g*_) and measure the leak current between the two electrodes. Figure 1B shows the leak current versus *U*_*g*_ from a test sample. Within *U*_*g*_ of −50 ∼ +50 V, the leak current was small and almost increased linearly with *U*_*g*_, without any sudden jump indicating breakdown of the oxide film. We thus limited *U*_*g*_ between −50 and +50 V in the present study.

Prior to all SF measurements, the thin-film sample was calcinated in UV ozone (BZS250GF-TC) for ∼1 h, then soaked in nonchromix/H_2_SO_4_ mixture (4 g nonchromix/100 ml H_2_SO_4_) for ∼0.5 h and rinsed thoroughly with deionized water (18.2 MΩ cm, Thermo Scientific Barnstead MicroPure UV). Besides, the Teflon cell was ultrasonicated successively in acetone, ethanol, and deionized water. Ultrapure deionized water and deuterated water (Sigma Aldrich, 99.9 atom % D) were used in the experiment.

### Laser and Optical System

A regenerative amplifier (Spitfire, Spectra-Physics) seeded by a Ti:sapphire oscillator (MaiTai SP, Spectra-Physics) was used to produce about 7 W of 800 nm, 35 fs pulses at a 2 kHz repetition rate. Around 40% output beam was used to generate a broadband IR beam (∼500 cm^−1^) centered at about 3,300 cm^−1^ by pumping an TOPAS-C/DFG system (Spectra-Physics), while the rest of the output beam was used to generate a narrowband beam (NIR) of ∼3 nm bandwidth by passing through an interference filter (LL01-808-25, Semrock). The NIR and IR beams were then focused and overlapped at the silica/water interface with incident angles of 45° and 60° (in air), respectively. The generated SFG signal was detected by a spectrograph (Acton SP300i, Princeton Instruments) and CCD camera (PyLoN: 400BR eXcelon, Princeton Instruments). All SF experiments were conducted in the atmosphere and room temperature. The beam polarization combination used was SSP (S for the SF beam, S for NIR, and P for IR).

### Electrical System

The operation principle of the electrolyte-insulator-semiconductor junction was described elsewhere (Waleed Shinwari et al., 2007; Wang et al., 2019). In a simplified representation, the Si/SiO_2_/water EIS junction can be modelled as three capacitors in series [*C*_*Si*_, *C*_*ox*_ and *C*_*w*_ (water), see insert of Figure 1C], with the respective capacitances per unit area being *C*_*i*_ *∼ ε*_*0*_ *ε*_*ri*_ */d*_*i*_ (*i* = *Si, ox, w*). *ε*_*0*_ is the vacuum permittivity, *ε*_*ri*_ and *d*_*i*_ are the dielectric constant and effective thickness of the *i*th layer, respectively. The charge density on each plate of the capacitors can be estimated *via σ* ∼ *U*_*g*_ *C*_tot_ = ∆*ϕ*_*i*_ *C*_*i*_, from which the potential drop on the *i*th layer (∆*ϕ*_*i*_) could be obtained. The electrostatic field across each layer is then *E*_*i*_ ∼ ∆*ϕ*_*i*_/*d*_*i*_. Compared to our previous study (Wang et al., 2019), the SiO_2_ film in this case was much thicker, leading to a much smaller *C*_*ox*_. The potential drop was then mostly on the oxide film (∼64%) instead of mostly on Si (Wang et al., 2019), which caused the electrostatic field as well as the dielectric polarization in the oxide layer to be much greater. The large polarization further led to a high surface density of bound charges that distributed on both oxygen and silicon sites (Jackson, 1999). This is also different from the previous case, where all surface charges were assumed to result from the protonation/deprotonation reactions (Wang et al., 2019). With *C*_*Si*_ ∼ 0.065 *μ*F/cm^2^, *C*_*w*_ ∼ 12 *μ*F/cm^2^ [effective capacitances deduced from (Wang et al., 2019)], and *d*_*ox*_ = 100 nm, *ε*_*rSi*_ ∼ 12, *ε*_*rox*_ = 3.9, and *ε*_*rw*_ ∼ 80, we estimated that at *U*_*g*_ ∼ 50 V, the total surface charge density on oxide is ∼0.07 e/nm^2^ (e refers to the elementary charge), and the electrostatic field within the interfacial water layer is about 4–16 mV/nm (between the two extreme cases when all charges are assumed to be reactive or bounded). Both were much greater than those achieved before, thus allowed us to investigate a much broader range of phenomena.

### Basics of SFVS

The basic principle of SFG was described elsewhere (Tian and Shen, 2014). As in a typical SFVS experiment, an infrared (IR) and a near infrared (NIR) beam are overlapped at the interface and generate a sum-frequency (SF) signal with a frequency of *ω*_*SF*_ *= ω*_*NIR*_ *+ ω*_*IR*_. The total SF signal intensity is:

$$I_{SF}\propto {\left|{\overset{↔}{\chi }}_{eff}^{\left(2\right)}:\vec{E}\left(\omega_{NIR}\right)\vec{E}\left(\omega_{IR}\right)\right|}^{2}$$ (1)

where ${\overset{↔}{\chi }}_{eff}^{\left(2\right)}$ is the effective second-order nonlinear susceptibility tensor of the system, and $\vec{E}$’s are the electric field vectors at the corresponding frequency. In our measurements in the OH stretching vibrational frequency range, the $\chi_{eff}^{\left(2\right)}$ was contributed from both the resonant part from interfacial OH bonds, as well as the non-resonant (NR) part from Si SCR and elsewhere in the Si/SiO_2_/water junction (Liu et al., 2019; Wang et al., 2019), which could be expressed as

$$\chi_{eff}^{\left(2\right)}=\chi_{R}^{\left(2\right)}+\chi_{NR}^{\left(2\right)},\chi_{R}^{\left(2\right)}=\underset{q}{\sum }\frac{A_{q}}{\omega -\omega_{q}+i\Gamma_{q}}$$ (2)

with *A*_*q*_, *ω*_*q*_, *Γ*_*q*_ corresponding to the amplitude, frequency and the damping coefficient of the *q*th resonance mode, respectively.

## Results and Discussion

### SFVS in D_2_O as References

As mentioned above, the total SF response consisted of both non-resonant and resonant contributions. For references, we first filled the cell with D_2_O and took SF spectra in the OH stretching vibrational range. Since D_2_O yields negligible signal within this range (Nihonyanagi et al., 2011; Myalitsin et al., 2016), we could therefore single out the non-resonant response (Wang et al., 2019). Figure 1C presented the gate-dependent, spectrally-integrated SF signal with D_2_O. In the previous study, for *U*_*g*_ between −1 and +1 V, the integrated non-resonant signal exhibited a quadratic dependence on *U*_*g*_ as expected for the electric-field-induced response from Si SCR (Lüpke, 1999; Wang et al., 2019), which almost vanished near the flat-band potential, and grew symmetrically around it (Wang et al., 2019). Yet here, within a much greater potential range between −50 and +50 V, the non-resonant SF signal was no longer quadratic, nor symmetric, with respect to *U*_*g*_ (Figure 1C) (Bian et al., 2018). The minimum still appeared near *U*_*g*_ ∼ 0, but the signal grew faster toward positive *U*_*g*_ compared to negative *U*_*g*_. This obvious deviation from a pure electric-field-induced behavior indicated that the non-resonant signal from other parts of the junction must be taken into accounts. Since the polarization of the SiO_2_ film and the charge density at SiO_2_/water interface are both much greater than those in the previous study, they can also contribute to the overall non-resonant response. We then attribute the deviation from quadratic response to the asymmetric interaction between the SiO_2_ surface and ionic species in the electrolyte at positive/negative *U*_*g*_. The water solution we used is weakly acidic (pH 5.8) due to the atmospheric carbon dioxide, so the major ionic species reacting with SiO_2_ are the positively charged hydroniums (H_3_O^+^/D_3_O^+^) or protons (H^+^/D^+^). At *U*_*g*_ < 0, the Si SCR, SiO_2_ film, and the SiO_2_/D_2_O interface were all polarized along the “upward” direction (defined to be pointing water to oxide) (Figure 1D). This led to a negative net charge density, including bound charges on bridging oxygen sites and SiO^−^ moieties. The latter could be protonated by above cations accumulating at the interface, thus partly compensated the overall polarization and the non-resonant signal strength. While at *U*_*g*_ > 0, the polarization of the SiO_2_ film was “downward” (Figure 1E), which resulted in a positive density of bound charges, most likely on silicon sites. The major anions (HCO_3_^−^/DCO_3_^−^ or CO_3_^2−^) attracted to the surface could not react with such silicon sites, and could not compensate for the surface charges as cations did. Combined this could cause the non-resonant signal to be weaker at *U*_*g*_ < 0, and stronger at *U*_*g*_ > 0.

### SFVS in H_2_O

**FIGURE 2:**
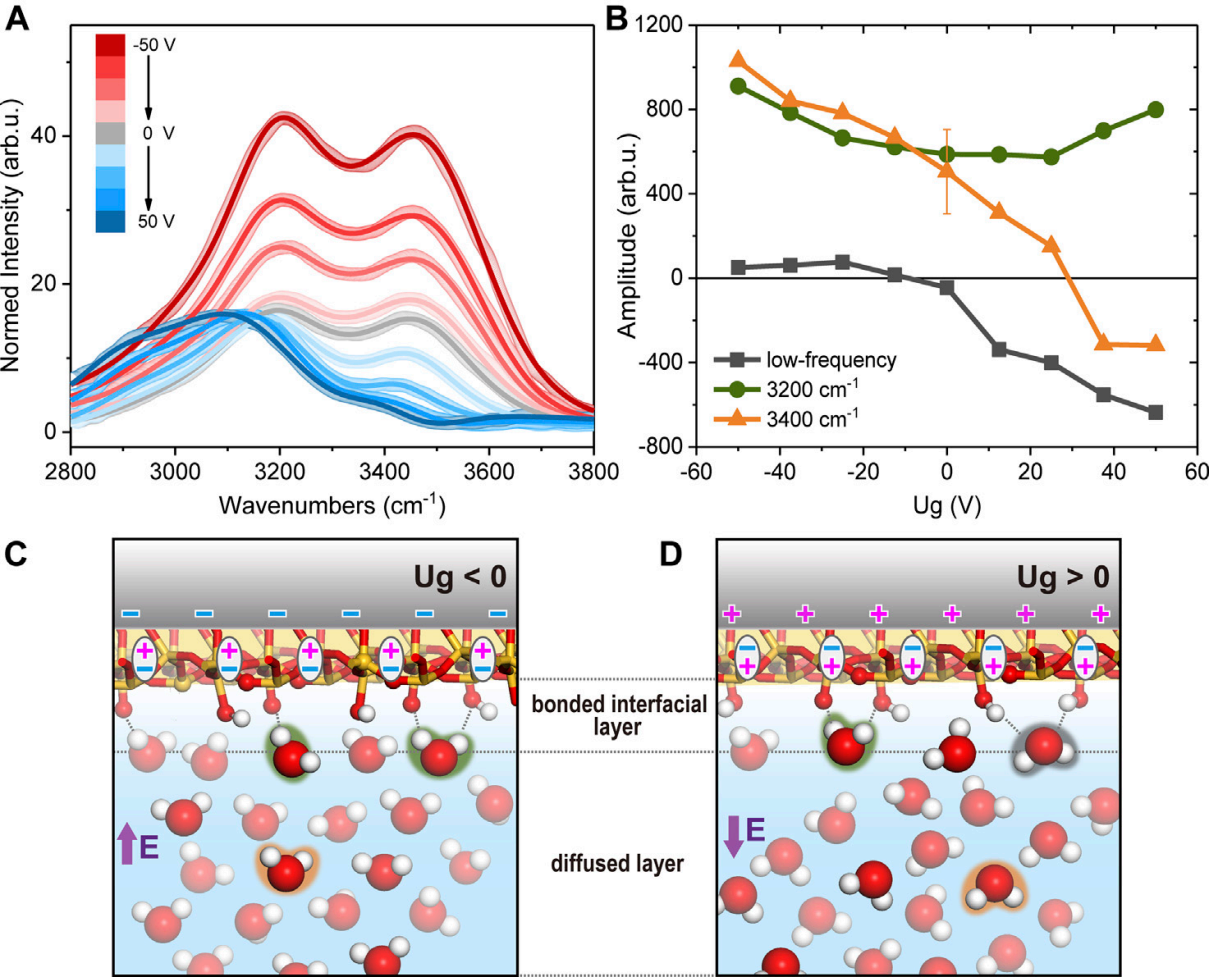
**(A)** Normalized SFVS spectra in H_2_O. Solid curves are fits. **(B)** Fitted amplitudes of the low frequency mode (black square), 3,200 cm^−1^ (green circle) and 3,400 cm^−1^ (orange triangle) modes. Sketches of the interfacial structures in H_2_O when **(C)** *U*_*g*_ *< 0* and **(D)** *U*_*g*_ *> 0*.

We then filled the cell with H_2_O and took OH spectra, which are presented in Figure 2A with varying *U*_*g*_. To correct for the variation in the infrared input energy, spectra were normalized to the intensity profile of non-resonant SiO_2_/D_2_O spectra. And since the non-resonant spectra profiles were not sensitive to the potential, we use the averaged spectra at all potentials for normalization to increase the signal-noise-ratio. In the previous study for *U*_*g*_ between −1 and +1 V, the non-resonant signal from Si SCR was comparable to the OH resonances from H_2_O, and spectra were from the interference between the two components (Wang et al., 2019). Here with a much thicker film, and at much greater *U*_*g*_, the spectra became dominated by OH resonances (Figures 1C, 2A). We first discuss the overall intensity variation of OH spectra versus potential. The integrated spectral intensity was the lowest at the most positive potential, grew slowly as approaching the neutral potential, but started to increase rapidly toward more negative potential (Figure 1C). Since the ionic composition remained unchanged in our case, so did the Debye length and beam coherence length, change in the spectral intensity directly reflected the degree of polar orientation of contributing OH moieties. From the features mentioned above, we concluded that the OH bonds were less (more) ordered when the surface was covered by positive (negative) bound charges. The overall trend agreed with that observed for the bulk-SiO_2_/H_2_O interfaces, that OH intensity is greater at higher pH value (Ostroverkhov et al., 2004; 2005; DeWalt-Kerian et al., 2017), as well as at lower ion concentrations (Jena and Hore, 2009; Yang et al., 2009; Jena et al., 2011; Li et al., 2015; Hore and Tyrode, 2019). Without extra buffer ions, the highest OH peak intensity recorded was near pH 11.5, and was roughly twice of that at pH 5.8 at the SiO_2_/H_2_O interface (Ostroverkhov et al., 2004; 2005; DeWalt-Kerian et al., 2017). While in our case, the peak intensity at *U*_*g*_ = −50 V was over three times greater than that at *U*_*g*_ = 0 V. The zero potential spectrum corresponded to the usual pH 5.8 spectrum for a bulk-SiO_2_/H_2_O interface (Wang et al., 2019). That means we achieved an even higher degree of polar orientation of interfacial OH bonds than that achievable at the bulk-SiO_2_/H_2_O interface. At pH 11.5, the SiO_2_ surface is often regarded as highly deprotonated with a surface charge density being ∼ −1.4 e/nm^2^; meanwhile, the Debye length is about 5.3 nm (without additional buffer ions), and the SF signal in the OH range is considered mostly from the topmost layer of water molecules directly adsorbed to the interface, or equivalently, in the bonded interfacial layer (Figures 2C,D) or topmost EDL layer (Urashima et al., 2018). In our system, at pH 5.8 and *U*_*g*_ = −50 V, the net charge density was lower than that at pH 11.5, but the Debye length is as long as 240 nm due to the low ion concentration (the beam coherence length was 50–60 nm). Therefore, the strong SF signal at negative *U*_*g*_ could contain a large contribution from water molecules in the diffuse layer (Figures 2C,D). According to the classical dynamic simulation, an electrostatic field as strong as 10^1^ mV/nm was needed to appreciably reorient the interfacial water molecules (Wang et al., 2019). The gating field in our case is strong enough to make the above scenario possible.

Before we go on with detailed analysis of OH spectra, we would like to emphasize again that in the present study with a thick silica film, a large portion of the surface charges was due to the polarization of oxide film. As we mentioned above, the distribution of bound charges is not necessarily the same with that from surface chemistry. For example, when pH is low, the positive charge centers are on SiOH_2_^+^ moieties at the bulk-silica/water interface due to protonation of SiOH species; while upon gating, positive surface charges could be simply due to the polarization of Si-O bonds. With only interfacial OH spectra, we could not distinguish between the two cases at this stage, thus only qualitative interpretations would be provided as follows. Here we discuss the overall variation in the spectral profile. Like those from bulk-SiO_2_/H_2_O interfaces, our spectra exhibited two major resonance features (Ostroverkhov et al., 2004; 2005; Myalitsin et al., 2016): the one at ∼3,200 cm^−1^ and another at ∼3,400 cm^−1^. The exact origin of the two peaks is still being investigated (Sovago et al., 2008; Myalitsin et al., 2016), but it is generally agreed that the lower the OH stretching frequency is, the stronger, and/or more ordered, H-bonding environment the corresponding moieties sense. On the bulk-SiO_2_/H_2_O interfaces, the 3,200 cm^−1^ mode (assigned to the topmost water layer (Urashima et al., 2018)) was found to grow more rapidly than the 3,400 cm^−1^ one (H-bonded to bulk-like water molecules (Montenegro et al., 2021)) at increasing pH, which suggested a more ordered water structure near the more negatively charged surface. In contrast, in our case, when the SiO_2_ surface bound charge evolved from positive to negative, the 3,400 cm^−1^ mode appeared to increase more rapidly. This agreed with our discussion above, that the polar ordered OH signal detected at strongly negative *U*_*g*_ had a large contribution from the diffuse layer composed of bulk liquid water, and thus exhibited a greater 3,400 cm^−1^ contribution.

Since there was no direct information about the spectral phase and exact field distribution in the EDL, we could not separate the double layer contributions as in Refs (Tian and Shen, 2014; Urashima et al., 2018). Therefore, we tentatively fitted the spectra in the conventional means by using Equations (1) and (2) (Figures 2A,B), which yielded reasonable results for qualitative understanding (Ostroverkhov et al., 2004; Myalitsin et al., 2016). Fitting parameters are also tabulated in Table 1. The fitting involved a low frequency mode ∼2,800–3,000 cm^−1^ besides the 3,200 cm^−1^ and 3,400 cm^−1^ modes, as in previous studies (Ostroverkhov et al., 2004; 2005; Nihonyanagi et al., 2011; Pezzotti et al., 2019; Wang et al., 2019; Rehl and Gibbs, 2021). When the surface charge varied from negative to positive, the 3,400 cm^−1^ mode changed from a large positive amplitude to a negative one. This corresponds to our discussion above, that water molecules inside diffuse layer made a major contribution to the 3,400 cm^−1^ peak and reoriented at varying *U*_*g*_. This also defined that the positive amplitude from fitting corresponded to an “upward” polarization. For the lowest frequency mode, it had a negligible amplitude at negative *U*_*g*_, and increased negatively toward more positive potential. This agreed with previous observations on neutral or positively charged SiO_2_ surfaces, and the mode could be attributed to “downward” OH groups accepting H-bonds with SiOH species (highlighted in black color in Figure 2D). The 3,200 cm^−1^ mode did not exhibit as much amplitude change as the other two modes, and remained positive in the whole potential range. We attributed this mode to “upward” OH groups donating H-bonds to the SiO_2_ surface (highlighted in green in Figures 2C,D), similarly to the findings in Refs (Urashima et al., 2018; Rehl and Gibbs, 2021). Combining the fitting results and discussions above, we summarized the microscopic pictures of the interface. At negative *U*_*g*_ (Figure 2C), the SiO_2_ film was polarized “upward”, and the surface held negative bound charges centered at oxygen sites. These sites could accept H-bonds from vicinal water molecules and caused an upward OH dipole orientation. Meanwhile, the gating field penetrating into the bulk water also rendered water molecules in diffuse layer to be aligned “upward”. The two polarizations superimposed constructively, leading to an enhancement of SF intensity at negative *U*_*g*_. And due to the long Debye length in our case, the SF intensity was even stronger than that achieved at pH 11.5 for a bulk-SiO_2_/H_2_O interface. At positive *U*_*g*_, the SiO_2_ film was polarized “downward”, and the surface held positive bound charges, presumably centered at silicon sites. Meanwhile, it was still the surface SiOH groups that form H-bonds with vicinal water molecules. As SiOH can be both H-bond donors and acceptors, they led to both upward (highlighted in green) and downward (highlighted in black) alignments of water OH groups as shown in Figure 2D. The former conflicted with the preferred downward field induced alignment (highlighted in orange) in diffuse layer (Figure 2D), and therefore the overall polar orientation was not as strong as that observed at negative *U*_*g*_.

**TABLE 1:** The fitting parameters of the normalized SFVS spectra in H_2_O.

*U*_*g*_ (V)	Low-frequency			3,200 cm^−1^			3,400 cm^−1^			$\chi_{NR}^{\left(2\right)}$	
	$\omega_{1}$ (cm^−1^)	A_1_ (a. u.)	$\Gamma_{1}$ (cm^−1^)	$\omega_{2}$ (cm^−1^)	A_2_ (a. u.)	$\Gamma_{2}$ (cm^−1^)	$\omega_{3}$ (cm^−1^)	A_3_ (a. u.)	$\Gamma_{3}$ (cm^−1^)	Re^a^ (a. u.)	Im^b^ (a. u.)
−50	3,008	51	107	3,187	911	155	3,477	1,030	225	−0.17	0.07
−37.5	2,999	61	106	3,176	785	150	3,492	840	204	−0.07	0.22
−25	3,012	76	116	3,174	665	144	3,489	783	206	−0.02	0.26
−12.5	2,983	15	116	3,180	622	158	3,464	666	223	−0.04	0.06
0	2,861	−45	111	3,186	587	161	3,459	505	209	−0.01	−0.25
12.5	2,862	−338	158	3,181	586	145	3,442	310	169	0.40	−0.22
25	2,877	−400	138	3,166	575	140	3,415	151	155	0.46	−0.28
37.5	2,890	−553	143	3,158	699	152	3,419	−314	151	0.68	−0.15
50	2,865	−636	136	3,125	799	179	3,398	−318	141	0.78	−0.35

a Re: the real part of the non-resonant background.

b Im: the imaginary part of the non-resonant background.

## Conclusion

To conclude, by applying an electrostatic gate potential to a silica thin film in neutral water, we observed a prominent enhancement of interfacial OH sum-frequency vibrational signals, with even greater relative intensity than that achieved at bulk-silica/water interfaces in strong basic solution. By fitting and analyzing the spectra, we attributed this observation to an effective field alignment of water molecules throughout the diffuse layer, which was resultant from the small number of ions and weak screening in our case. This demonstrates the unique tuning capability of the EIS structure as we previously reported (Wang et al., 2019). On the other hand, as we mentioned above, we could not distinguish between bound charges and surface charges from protonation/deprotonation reactions at this stage, and only qualitative interpretations were provided based on assumptions on the SiO_2_ surface structures. Further studies on the SiO_2_ side, for example the *in situ* detection of its surface phonon spectra in water, would be essential for us to fully understand the phenomenon and the entire aqueous interface at the molecular level.

## Data Availability

The original contributions presented in the study are included in the article/supplementary material, further inquiries can be directed to the corresponding author.
